# Motor Adaptation in Parkinson’s Disease During Prolonged Walking in Response to Corrective Acoustic Messages

**DOI:** 10.3389/fnagi.2019.00265

**Published:** 2019-09-24

**Authors:** Mattia Corzani, Alberto Ferrari, Pieter Ginis, Alice Nieuwboer, Lorenzo Chiari

**Affiliations:** ^1^Department of Electrical, Electronic, and Information Engineering, University of Bologna, Bologna, Italy; ^2^Department of Rehabilitation Sciences, Neurorehabilitation Research Group, KU Leuven, Leuven, Belgium

**Keywords:** Parkinson’s disease, motor adaptation, continuous gait, auditory cue, verbal feedback, wearable sensors

## Abstract

Wearable sensing technology is a new way to deliver corrective feedback. It is highly applicable to gait rehabilitation for persons with Parkinson’s disease (PD) because feedback potentially engages spared neural function. Our study characterizes participants’ motor adaptation to feedback signaling a deviation from their normal cadence during prolonged walking, providing insight into possible novel therapeutic devices for gait re-training. Twenty-eight persons with PD (15 with freezing, 13 without) and 13 age-matched healthy elderly (HE) walked for two 30-minute sessions. When their cadence varied, they heard either intelligent cueing (IntCue: bouts of ten beats indicating normal cadence) or intelligent feedback (IntFB: verbal instruction to increase or decrease cadence). We created a model that compares the effectiveness of the two conditions by quantifying the number of steps needed to return to the target cadence for every deviation. The model fits the short-term motor responses to the external step inputs (collected with wearable sensors). We found some significant difference in motor adaptation among groups and subgroups for the IntCue condition only. Both conditions were instead able to identify different types of responders among persons with PD, although showing opposite trends in their speed of adaptation. Increasing rather than decreasing the pace appeared to be more difficult for both groups. In fact, under IntFB the PD group required about seven steps to increase their cadence, whereas they only needed about three steps to decrease their cadence. However, it is important to note that this difference was not significant; perhaps future work could include more participants and/or more sessions, increasing the total number of deviations for analysis. Notably, a significant negative correlation, *r* = −0.57 (*p*-value = 0.008), was found between speed of adaptation and number of deviations during IntCue, but not during IntFB, suggesting that, for people who struggle with gait, such as those with PD, verbal instructions rather than metronome beats might be more effective at restoring normal cadence. Clinicians and biofeedback developers designing novel therapeutic devices could apply our findings to determine the optimal timing for corrective feedback, optimizing gait rehabilitation while minimizing the risk of cue-dependency.

## Introduction

Parkinson’s disease (PD) is a neurodegenerative disorder predominantly characterized by the depletion of dopamine and dopaminergic neurons in the basal ganglia (BG) (Mazzoni et al., 2012). The disease affects different neural networks and neurotransmitters, leading to impaired ability to learn and express automatic actions, such as walking (Redgrave et al., 2010). The use of external sensory cues (e.g., auditory, visual) to reinforce attention toward the task (Lee et al., 2012) is an effective gait-rehabilitation strategy for persons with PD; the cues stimulate the executive voluntary component of action (Morris, 2006; Morris et al., 2008; Ferrazzoli et al., 2018) by activating the attentional-executive motor control system and bypassing the dysfunctional, habitual, sensorimotor BG network (Morris, 2006; Morris et al., 2008, 2010; Redgrave et al., 2010; Shine et al., 2014; Tard et al., 2015; Arnulfo et al., 2018; Pozzi et al., 2019). This strategy helps people with PD improve gait consistency and rhythmicity. In the past, auditory cueing during gait has typically been provided continuously in an open loop (regardless of gait performance). However, continuous cueing may result in cue-dependency and habituation on external stimuli (Nieuwboer et al., 2009; Spildooren et al., 2012; Vercruysse et al., 2012; Bohnen and Jahn, 2013).

One of the most innovative developments in the quantitative assessment and management of PD symptoms is the use of wearable technologies during gait (Sánchez-Ferro and Maetzler, 2016), which are able to provide customized cueing: stimuli are triggered when gait deviates from normal, thus providing patients with immediate feedback on their performance. These closed-loop stimuli [audio (Ginis et al., 2016; Ginis et al., 2017a, b), visual (Ahn et al., 2017; Chong et al., 2011), audio-visual, (Espay et al., 2010) or proprioceptive (Mancini et al., 2018)] are known as intelligent inputs (Ginis et al., 2017a, b). In contrast to open-loop systems, in closed-loop systems the external information does not necessarily become part of the participants’ movement representation (as explained by the “guidance hypothesis”), thus possibly decreasing the development of cue-dependency (Nieuwboer et al., 2009). Wearable systems also permit data collection in a more naturalistic environment (Espay et al., 2010; Ginis et al., 2016).

**FIGURE 1:**
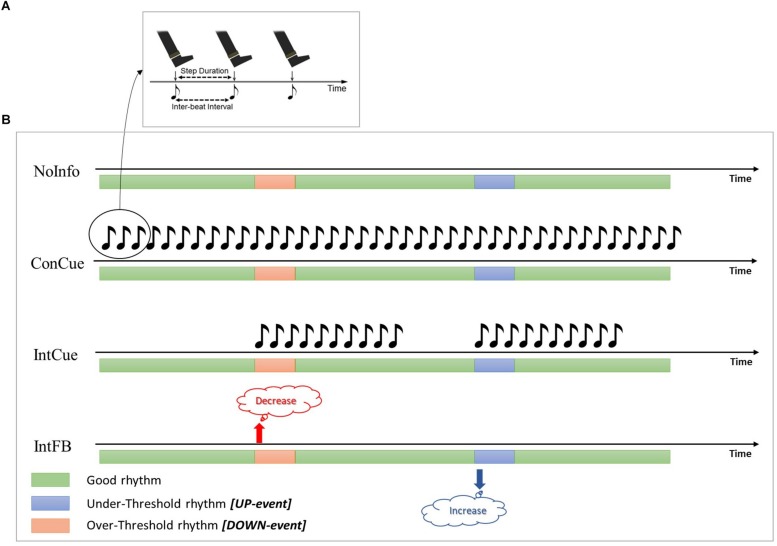
**(A)** During ConCue and IntCue the participants were instructed to follow the rhythm by stepping to the beat of the metronome set at the mean cadence of the reference walk. **(B)** Schematic representations of the different intelligent inputs used in the protocol. Green, blue and red bars represent the periods during which the cadence is good, deviates below the threshold, or deviates above the threshold, respectively. *NoInfo***:** no external information was given during the entire walk; *ConCue***:** during the entire walk, participants received the auditory rhythm set at the mean cadence of the reference walk; *IntCue*: participants received the same auditory rhythm as in *ConCue*, but only for ten beats and only when the cadence deviated from the reference cadence; *IntFB*: participants received verbal feedback to “Increase the rhythm” or “Decrease the rhythm” when the cadence was more than 5% slower or faster (respectively) than the reference cadence.

Two previous studies (Ginis et al., 2017a, b) compared the effects of intelligent auditory cueing (IntCue) and intelligent verbal feedback (IntFB) on gait as alternatives to traditional open-loop continuous cueing (ConCue) (see Figure 1). Those studies showed that both IntCue and IntFB conditions were at least as effective as ConCue for optimizing gait in PD. For example, the first study showed that IntFB was most effective at maintaining normal cadence at the end of a 30-minute-long gait exercise, although it also increased the gait variability (deviations from the target pace) in persons with PD compared to healthy controls. Furthermore, during IntCue, the number of deviations was actually smaller than during the no-input condition in PD (Ginis et al., 2017b).

In the second one, persons with and without freezing of gait (FOG+ and FOG-) were compared (Ginis et al., 2017a). The results show that the FOG+ group benefits less from intelligent inputs than the FOG- group, probably due to more affected motor and cognitive functions (Ginis et al., 2017a). In addition, the former had significantly more gait deviations than the latter during IntCue and IntFB conditions, but not when continuously cued. Although these findings suggest that ConCue was more effective in supporting prolonged gait in the FOG+ group, the majority of these persons favored the IntFB condition (Ginis et al., 2017a).

These two works (Ginis et al., 2017a, b) adopted a macro approach to analyze the effect of wearable sensors and external inputs on continuous gait in PD: they did not quantify the individual motor responses to the corrective messages. However, since many factors are at play during a prolonged walking trial, such as fatigue and learning, a micro-analysis is more appropriate, because it quantifies the motor adaptations during the participants’ immediate response to the IntFB and IntCue conditions. Thus, the cadence of the subjects’ first steps following each corrective acoustic message can be quantified.

The aim of this study is to characterize motor adaptation in response to corrective acoustic messages during prolonged walking in order to gain insight on how to better design novel therapeutic devices for gait re-training in PD (FOG- and FOG+). To this end we propose a new model for fitting the short-term motor responses to external inputs (collected with wearable sensors). Using this model we determine the number of steps needed to adapt gait pattern following corrective acoustic messages. We investigated adaptation speed during IntFB and IntCue conditions for the following groups: healthy elderly (HEg), persons with PD (PDg), and PD subgroups with (FOG+g) and without (FOG-g) freezing of gait. We hypothesized that IntFB would lead to a more effective adaptation than IntCue, due to its verbal nature. In fact, the IntFB has an explicit nature with a clear direction of change to adapt the gait, compared to IntCue, which requires some processing time to elaborate the direction of adaptation leading to a delay in the motor response. Furthermore, because persons with PD struggle to maintain normal gait (Hausdorff, 2009), we expected that slowing down would be easier than speeding up. For both conditions, they would adapt more quickly when they were directed to slow back down to their reference cadence (because they had speed up) than when they were directed to speed up (because they had slowed down).

To assess the effect of the clinical characteristics of the participants, we investigated the relationship that links motor adaptation with their disease severity and their cognitive status. Furthermore, to match our micro-analysis with the macro results of previous work (Ginis et al., 2017a,b), we also evaluated the relationship between motor adaptation speed and the number of deviations of each subject, to determine if persons who struggled more to walk consistently were slower to adapt to the corrective stimuli.

## Materials and Methods

The present study consists of a sub-analysis of another study which compared persons with PD to age-matched healthy subjects on several gait characteristics throughout 30 min of walking during four different auditory input conditions (Ginis et al., 2017b). These sections briefly describe the participants and protocol of the previous study before presenting the motor adaptation model and statistical analysis.

### Original Study – Participants, Protocol, and Materials

Twenty-eight persons with PD were recruited from the Movement Disorders clinic of the University Hospitals Leuven based on the following inclusion criteria: (Mazzoni et al., 2012) idiopathic PD, diagnosed according to the United Kingdom Brain Bank criteria; (Redgrave et al., 2010) Hoehn and Yahr stage I–III; and (Lee et al., 2012) stable PD medication for the past month and anticipated for the following 2 months. Exclusion criteria were: (Mazzoni et al., 2012) cognitive deficits (Mini Mental State Examination <24/30); (Redgrave et al., 2010) subjectively unable to walk unassisted for 30 min; (Lee et al., 2012) fluctuating response to levodopa; (Morris, 2006) musculoskeletal or neurological conditions other than PD affecting gait; and (Morris et al., 2008) severe hearing problems precluding headphone use for auditory information. Participants were categorized into freezers, FOG+ (*n* = 15), and non-freezers, FOG- (*n* = 13), based on a score of one or higher on the New Freezing of Gait Questionnaire (NFOG-Q). All persons with PD were tested in their subjective ON-medication state, an average of 1 h after medication intake. It is important to note that no freezing episodes occurred during the study.

Thirteen age-matched HE were recruited from a database of voluntary study participants. The study design and protocol were approved by the local Ethics Committee of the KU Leuven and performed in accordance with the requirements of the International Council of Harmonization (Declaration of Helsinki, 1964). Written informed consent was obtained from each participant prior to the experiment. Over a period of 6 weeks, participants performed four 30-minute walks, with at least one week between walks, around an elliptical track measuring 24 m by 9 m. Prior to each 30-minute walk, the reference walk consisted of a fixed duration of a 1-min walk at a comfortable pace was recorded, to obtain the reference cadence. Participants started the 30-minute walk randomly in a clockwise or anti-clockwise direction, after which the starting direction was kept identical per person over the four sessions. After 15 min of walking, participants changed their walking direction (by crossing the trajectory diagonally) to counteract possible effects of disease dominance. In a randomized order, participants experienced one of the following conditions for the entire 30-minute walk: (Mazzoni et al., 2012) continuous cueing (ConCue); (Redgrave et al., 2010) intelligent cueing (IntCue); (Lee et al., 2012) intelligent feedback (IntFB); and (Morris, 2006) no information (NoInfo). Cueing and feedback were provided by an adaptive wearable system (Casamassima et al., 2014) through headphones (Sennheiser RS160, Sennheiser, Germany). During IntCue, for every deviation, participants received an auditory rhythm, consisting of ten beats at the reference cadence—whether it was a DOWN event (cadence over the threshold) or an UP event (cadence below the threshold). The threshold which triggered the stimulus was set as a variation of more than 5% from the reference cadence, calculated from the mean cadence of five consecutive steps. During IntFB, participants received a verbal instruction to “increase rhythm” or “decrease rhythm” based on the same criteria as during IntCue. The values for the IntCue and IntFB settings, as well as the duration of the 1-min reference walk, were based on user acceptability, determined during pilot testing prior to the study. All the conditions are shown in Figure 1. All walks were performed in the same hall at the same time and day of the week to minimize the effects of time and PD medication. Demographic information and clinical test results were collected: in particular, the Movement Disorders Society Unified Parkinson’s Disease Rating Scale—Motor Part (MDS-UPDRS III) (Goetz et al., 2008), Scale for Outcomes in Parkinson’s Disease-Cognition (SCOPA-Cog) (Marinus et al., 2003), and Montreal Cognitive Assessment (MoCA) (Gill et al., 2008). All the clinical tests were collected during the ON phase of medication before the start of the walking task to avoid potential influence of fatigue. Clinical tests were evenly distributed over the different assessment days.

Participants wore two foot-mounted inertial measurement units (IMUs) attached to the tops of their shoes using Velcro straps. The IMUs (EXLs1, EXEL srl, Italy) contained a tri-axial accelerometer, gyroscope, and magnetometer, sampled at 100 Hz and wirelessly streaming via Bluetooth to a computer. A custom Matlab (Mathworks Inc., United States) software application, using the algorithms currently implemented in the commercially available system Gait Tutor (mHealth Technologies, IT), processed the signals in real time during each 30-minute walk. The algorithm (validated for PD (Ferrari et al., 2016; Ginis et al., 2016) and described elsewhere (Casamassima et al., 2014)) computed cadence from the raw IMU data and registered any deviations from the pre-recorded reference cadence.

### Motor Adaptation Model

This section describes the analysis we performed for IntCue and IntFB conditions in order to evaluate motor adaptation after intelligent inputs.

Cadence for all conditions was calculated by combining the data from both feet. The system in the original study only retained the average cadence for every five steps of each foot, while to obtain better sensitivity we recreated the original cadence for every trial.

Next, adaptation (in response to both UP and DOWN events) was quantified by fitting a single-term exponential model (Eq. 1) to the cadence of the ten steps following a deviation. In Eq. (1), *y* is the fitted cadence expressed as a percentage of the difference with respect to the reference cadence, *k* is the exponential decay/growth rate [step^–1^], *x* is the number of steps after intelligent input (from 0 to 9), and *M* is the under-/over-threshold value [%].

(1) $$y=\pm M\,e^{-k\,x}$$

**FIGURE 2:**
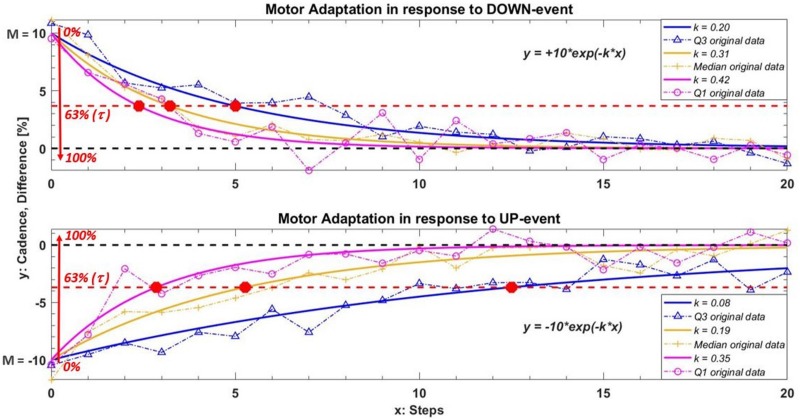
Example of the model used to quantify motor adaption in response to DOWN and UP events. In this example the under/over threshold value *M* is ± 10 and we used three different values of *k* to better understand the role of this parameter. The curves in orange, blue and magenta represent the median and relative interquartile values (Q3 in blue; Q1 in magenta) of the exponential fitting model obtained among all participants during IntFB condition. The orange plus, the blue triangles and the magenta circles are an example of the original cadence with the same *k* adaptation rate in one subject. The red circle in the graph show the relative τ value of each curves. This value indicates the steps needed to adapt gait pattern increasing/decreasing *M* of 63% toward the reference cadence following a corrective feedback. A higher value of *k* (or a smaller value of τ) corresponds to a faster motor adaptation.

The primary outcome was the exponential decay/growth rate *k* estimated for each UP or DOWN deviation (for all participants). Figure 2 shows a representation of the mathematical model in response to both DOWN and UP events. To better illustrate the role of *k*, each graph in Figure 2 reports three responses with different *k* values.

A higher value of *k* corresponds to a faster adaptation, as is clear in Figure 2. Next, we evaluated the relative step constant (intrinsic to an exponential decay/growth model), τ = *1 / k*, which (as shown in the graph) intercepts the curve at a value for *M* of 63%. This characteristic parameter is defined in our study as the number of steps required to reduce *M* sufficiently that participants are in the correct range. (Note that this percentage supposes a reasonable *M*). Therefore, τ represents what we can call a *refractory period*: the number of steps needed to bring the cadence back within the reference range following verbal/acoustic feedback, during which providing a new corrective message may have no effect.

### Statistical Analysis

A preliminary qualitative analysis compared the average responses to corrective stimuli between PDg, HEg, and FOG-g, FOG+g. We used our fitting model to quantify motor adaptation in terms of *k*, the exponential decay/growth rate during all the corrective acoustic messages received by the participants. We calculated the absolute median values and the relative interquartile range among all groups and subgroups.

Next, we used paired non-parametric statistics (Wilcoxon Signed Rank test) to evaluate the *condition effect* (IntCue vs. IntFB) and the *task effect* (UP event vs. DOWN event), analyzing differences in the average *k* rate of each subject only within the PDg, due to the small sample available. However, the resulting average differences do not say anything about specific motor responses (Rispens et al., 2015). On the other hand, situations where people show a high level of motor adaptation reflect their best possible performance and are thus of specific therapeutic interest. To identify the best performances, we investigated the 90th-percentile values of each subject in addition to the average *k* rate. Clearly, a paired test requires subjects who had corrective messages in both conditions or in both tasks (depending on the analysis).

Unpaired non-parametric statistics (Mann-Whitney *U* tests) were used to examine differences in the value of *k* between groups HEg and PDg (*group effect)* and subgroups FOG+g and FOG-g (*subgroup effect)*. In addition, we performed an exploratory analysis to assess whether subjects who had only UP events responded differently than those who had both UP and DOWN events. We assumed that for those subjects who tend to slow down, it may be more difficult to increase their rhythm in response to corrective acoustic messages—compared to subjects who tend to both slow down and speed up.

The relations between adaptation speed and clinical data were explored by correlating the participants’ scores on SCOPA-Cog and MoCA (cognitive aspect) and MDS-UPDRS III (disease severity) with their median *k* rate using Spearman rank correlation coefficients. The median *k* rate was also correlated with the number of deviations for each subject. Matlab (Mathworks Inc., United States) was used for all statistical analyses, with α = 0.05.

## Results

### Demographics

For simplicity, the demographic analysis (available from previous studies) is reported in Table 1 (Ginis et al., 2017a, b). PDg and HEg were well matched for age, body height, body weight, cognitive ability (MoCA), total self-reported daily physical activity (LAPAQ Total), and self-reported daily walking time (LAPAQ Walking). The PD group had significantly lower cognitive scores (SCOPA-Cog). Freezers (FOG+) and non-freezers (FOG-) were well matched for age, body morphology (weight, height, and leg length), self-reported daily walking, and total daily activity time (LAPAQ), as well as for Hoehn and Yahr stage. The FOG+g had a significantly longer disease duration, lower cognitive scores (MoCA), and more reported gait difficulties on the 12-item gait scale (12G) than the FOG-g.

**TABLE 1:** Results are reported as mean (standard deviation) in the case of parametric statistics and as median (quartile 1– quartile 3) in the case of non-parametric statistics.

	**PD (*n* = 28)**	**HE (*n* = 13)**	**Sign.**	**FOG+ (*n* = 15)**	**FOG− (*n* = 13)**	**Significant**
Age (years)	62.04 (6.91)	60.23 (6.07)	*p = 0.42*	62.80 (6.91)	61.15 (7.08)	*p = 0.54*
Gender (M/F)^a^	23/5	7/6	*p = 0.07*	14/1	9/4	*p = 0.09*
Body weight (kg)	82.73 (15.83)	74.39 (14.63)	*p = 0.12*	79.93 (14.56)	85.95 (17.20)	*p = 0.33*
Body height (cm)	174.00 (8.37)	169.85 (7.99)	*p = 0.14*	173.07 (5.61)	175.08 (10.89)	*p = 0.56*
Leg length left (cm)	92.54 (5.99)	90.15 (4.20)	*p = 0.21*	92.13 (3.72)	93.00 (8.01)	*p = 0.73*
Leg length right (cm)	92.14 (5.77)	90.46 (4.35)	*p = 0.36*	91.80 (3.26)	92.54 (7.88)	*p = 0.76*
Disease duration (years)	10.57 (6.71)	/	/	13.20 (5.55)	7.54 (6.84)	***p = 0.03***
H and Y (1/2/2.5/3)^a^	1/12/7/7	/	/	0/6/4/5	1/7/3/2	*p = 0.14*
MDS-UPDRS III (0–132)	34.57 (14.37)	/	/	37.93 (14.39)	30.69 (13.88)	*p = 0.19*
LEDD (mg/24 h)	517.42 (312.97)	/	/	622.98 (338.51)	395.62 (238.12)	*p = 0.05*
MoCA (0–30)	26.36 (2.18)	27.46 (2.22)	*p = 0.14*	25.27 (2.15)	27.62 (1.45)	***p = 0.003***
SCOPA-Cog (0–42)^b^	29.50 (26.00–31.25)	34.00 (32.00–35.00)	***p = 0.001***	29.00 (22.25–30.00)	31.00 (27–31.25)	*p = 0.29*
LAPAQ walking (min/day)^b^	14 (5–30)	11 (7–21)	*p = 0.71*	8.57 (0.89–32.86)	15.00 (6.43–20.00)	*p = 0.53*
LAPAQ total (min/day)^b^	127 (56–198)	207 (105–326)	*p = 0.14*	117.14 (67.14–181.07)	136.43 (52.14–322.86)	*p = 0.86*
12 G (0–87)^b^	9.50 (5.75–14.50)	0 (0–0)	***p < 0.001***	13.00 (9.50–19.50)	6.00 (3.00–9.00)	***p = 0.007***

*Bold numbers indicate significant differences between groups and subgroups. MDS-UPDRS III Movement Disorders Society Unified Parkinson Disease Rating Scale-Motor Part, *LEDD* levodopa equivalent daily dosage, *MoCA* Montreal Cognitive Assessment, *LAPAQ* LASA Physical Activity Questionnaire, *12G* 12 item gait scale, *SCOPA-Cog* Scale for Outcomes in Parkinson’s Disease-Cognition. All the clinical tests were collected during the ON phase. ^*a*^ Chi-squared statistics. ^*b*^ Non-parametric statistics were applied.*

### Qualitative Adaptation Plots

**FIGURE 3:**
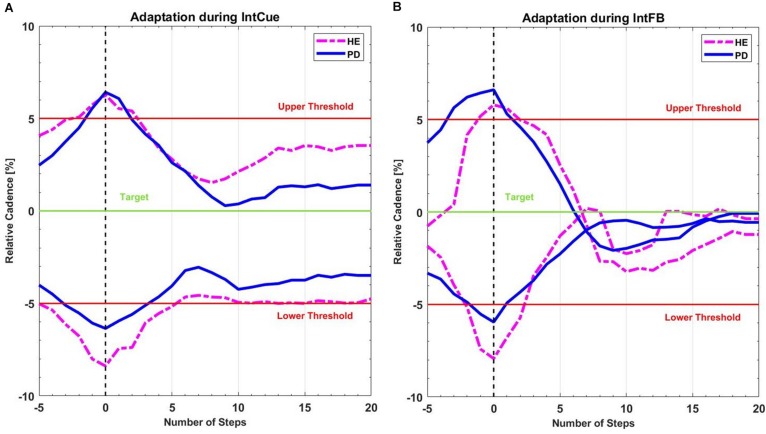
Mean recreated cadence of all PDg (full lines in blue) and all HEg (dashed lines in magenta) after all corrective acoustic messages **(A)** during IntCue and **(B)** during IntFB condition, starting 5 steps before until 20 steps after the deviation. The vertical black dashed line represents the onset of corrective input. The green target line represents the stepping rhythm recorded during the 1-min reference walk. The red lines mark the 5% deviation levels above and below the target line.

In this preliminary analysis we qualitatively highlighted the average behavior of the participants following the corrective acoustic messages. We compared the average cadence between HEg and PDg and subgroups FOG+g and FOG-g, from five steps before the deviation until 20 steps after. Figure 3 shows the average original cadence responses to all corrective acoustic messages received by HEg and PDg during both conditions.

**FIGURE 4:**
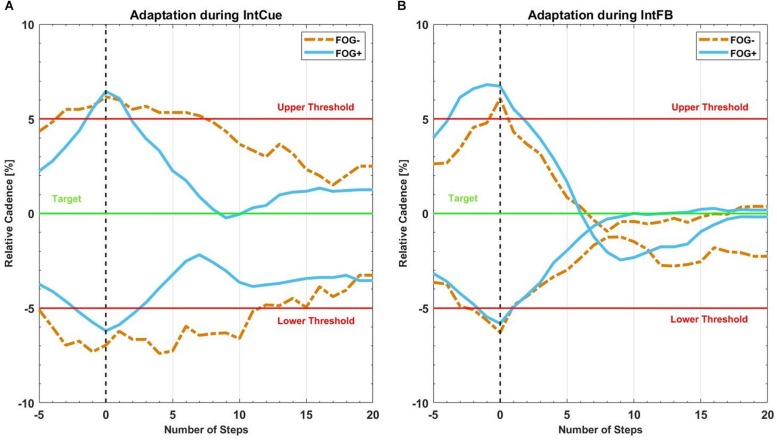
Mean recreated cadence of all FOG+g (full lines in light blue) and all FOG-g (dashed lines in yellow) after all corrective acoustic messages, **(A)** during IntCue and **(B)** during IntFB condition, from five steps before to 20 steps after the deviation. The vertical black dashed line represents the corrective input onset. The green target line represents the stepping rhythm recorded during the 1-min reference walk. The red upper and lower thresholds mark the 5% deviation levels above and below the target line.

Figure 4 reports the same analysis as Figure 3, comparing FOG- and FOG+ subgroups in both conditions.

### Number of Deviations

To improve interpretability and readability of our analysis, in Table 2 we reported the total number of deviations, already presented and discussed in the original study (Ginis et al., 2017a, b).

**TABLE 2:** Total number of deviations received by the groups in response to UP **(A)** and DOWN **(B)** messages.

**A**		

***Deviations UP-event***	**IntCue**	**IntFB**
HE	54	9
PD	119	139
FOG+	95	96
FOG-	24	43

**B**		
***Deviations DOWN-event***	**IntCue**	**IntFB**
HE	13	6
PD	62	88
FOG+	54	81
FOG-	8	7

**IntCue*, intelligent auditory cueing; *IntFB*, intelligent verbal feedback; *UP event*, cadence under the threshold – Increase rhythm; *DOWN event*, cadence over the threshold – Decrease rhythm; *PD*, people with Parkinson’s disease; *HE*, healthy elderly; *FOG+*, people with Parkinson’s disease with freezing of gait; *FOG-*, people with Parkinson’s disease without freezing of gait.*

### Condition Effect: IntCue vs. IntFB

As can be seen in Table 3, although the fastest median *k* rate (i.e., larger median *k* rate) occurred during IntFB, the differences in *k* rates for the two types of conditions were not significant for PDg. It is important to note that, to perform a paired analysis, we had to exclude some subjects: only 12 of the 28 PD subjects received at least one UP message during the two conditions and only four subjects received at least one DOWN message.

**TABLE 3:** Condition effect. Index of adaptation *k* is reported as median (quartile 1– quartile 3) among all groups in response to UP **(A)** and DOWN **(B)** messages.

**A**				

***k* UP-event**	**IntCue**	**IntFB**	**Mean Condition effect**	**90th Condition effect**
HE	0.06 (0.00–0.10)	0.21 (0.18–0.38)	–	–
#deviators	#5	#6		
PD	0.13 (0.06–0.18)	0.15 (0.07–0.35)	*p* = 0.260	*p* = 0.151
#deviators	#14	#16	(*n* = 12)	(*n* = 12)
FOG+	0.13 (0.08–0.18)	0.20 (0.10–0.36)	–	–
#deviators	#10	#11	(*n* = 9)	(*n* = 9)
FOG-	0.08 (−0.02–0.18)	0.12 (0.05–0.30)	–	–
#deviators	#4	#5		

**B**				
***k* DOWN-event**	**IntCue**	**IntFB**	**Mean Condition effect**	**90th Condition effect**
HE	0.23 (0.18–0.26)	0.28 (0.22–0.35)	–	–
#deviators	#2	#3		
PD	0.21 (0.07–0.44)	0.33 (0.19–0.45)	*p* = 0.250	*p* = 0.625
#deviators	#8	#11	(*n* = 4)	(*n* = 4)
FOG+	0.24 (0.09–0.49)	0.31 (0.18–0.45)	–	–
#deviators	#6	#7	(*n* = 3)	(*n* = 3)
FOG-	0.01 (−0.01–0.06)	0.36 (0.24–0.44)	–	–
#deviators	#2	#4		

**IntCue*, intelligent auditory cueing; *IntFB*, intelligent verbal feedback; *UP event*, cadence under the threshold – Increase rhythm; *DOWN event*, cadence over the threshold – Decrease rhythm; *#deviators* participants who have at least one deviation; *PD*, people with Parkinson’s disease; *HE*, healthy elderly; *FOG+*, people with Parkinson’s disease with freezing of gait; *FOG-*, people with Parkinson’s disease without freezing of gait; *Mean Condition effect*, paired analysis using the average *k* rate of each subject; *90th Condition effect*, paired analysis using the 90th percentile value *k* rate of each subject. *n*, number of participants included in the paired-analysis.*

Next, a subgroup evaluation was carried out (see Table 3). No statistical analysis was performed for HEg, FOG+g or FOG-g, due to the small number of paired samples, which would increase the possibility of a type II error. In line with the PDg findings, this analysis suggested a slightly faster adaptation during IntFB than IntCue looking at the absolute median value.

### Group Effect: PDg vs. HEg; SubGroup Effect: FOG+g vs. FOG-g

Differences in motor adaptation between the groups (HEg vs. PDg) and subgroups (FOG+g vs. FOG-g) were analyzed during IntFB (Tables 4A-1, A-2) and IntCue conditions (Tables 4B-1, B-2). During the IntCue condition, PDg had a significantly faster adaptation than HEg in response to the UP event (*p-*value < 0.000) and FOG+g adapted significantly faster than FOG-g in response to the DOWN event (*p-*value = 0.006).

**TABLE 4:** Task and group effect. Index of adaptation *k* is reported as median (quartile 1– quartile 3) among all groups for (A-1, A-2) IntFB condition, (B-1, B-2) for IntCue condition.

**A-1**					**A-2**			
	
***k* IntFB**	**UP-event**	**DOWN-event**	**Mean Task effect**	**90th Task effect**	***k* IntFB**	**UP-event**	**DOWN-event**	**Task effect**
HE	0.21 (0.18–0.38)	0.28 (0.22–0.35)	–	–	FOG+	0.20 (0.10–0.36)	0.31 (0.18–0.45)	–
#deviators	#5	#3			#deviators	#11	#7	(*n* = 5)
PD	0.15 (0.07–0.35)	0.33 (0.19–0.45)	*p* = 0.630	*p* = 0.195	FOG-	0.12 (0.05–0.30)	0.36 (0.24–0.44)	–
#deviators	#16	#11	(*n* = 8)	(*n* = 8)	#deviators	#5	#4	
**Group effect**	*p* = 0.255	*p* = 0.734			**SubGroup effect**	*p* = 0.101	*p* = 0.460	

**B-1**					**B-2**			
	
***k* IntCue**	**UP-event**	**DOWN-event**	**Mean Task effect**	**90th Task effect**	***k* IntCue**	**UP-event**	**DOWN-event**	**Task effect**
HE	0.06 (0.00–0.10)	0.23 (0.18–0.26)	–	–	FOG+	0.13 (0.08–0.18)	0.24 (0.09–0.49)	–
#deviators	#6	#2			#deviators	#10	#6	(*n* = 4)
PD	0.13 (0.06–0.18)	0.21 (0.07–0.44)	*p* = 0.156	*p* = 0.312	FOG-	0.08 (−0.02–0.18)	0.01 (−0.01–0.06)	–
#deviators	#14	#8	(*n* = 5)	(*n* = 5)	#deviators	#4	#2	
**Group effect**	***p* < 0.000**	*p* = 0.872			**SubGroup effect**	*p* = 0.073	***p* = 0.006**	

**IntCue* intelligent auditory cueing; *IntFB* intelligent verbal feedback; *#deviators* participants who have at least one deviation; *PD* people with Parkinson’s disease; *HE* healthy elderly; *FOG+* people with Parkinson’s disease with freezing of gait; *FOG-* people with Parkinson’s disease without freezing of gait; *Mean Task effect* paired analysis using the average *k* rate of each subject; *90th Task effect* paired analysis using the 90th percentile value *k* rate of each subject. *Group effect* unpaired analysis between HE and PD groups. *SubGroup effect* unpaired analysis between FOG+ and FOG- groups. Bold numbers indicate significant differences in the task (UP vs. DOWN event), groups (PD vs. HE) and subgroups effect (FOG+ vs. FOG-). *n*, number of participants included in the paired-analysis.*

### Task Effect: UP Event vs. DOWN Event

Although the fastest median *k* rate occurred following a DOWN event, the differences in *k* rates for the two types of event were not significant for PDg (Tables 4A-1, B-1). This finding also holds true within the HEg, FOG+g and FOG-g (Table 4), but here no statistical analysis was performed because of the small number of paired samples. Moreover, only eight persons with PD experienced at least one of each event type during the IntFB condition and only five during the IntCue condition.

We performed an exploratory analysis to assess whether members of PDg who had only UP events responded differently than those who experienced both UP and DOWN events. The results indicate a different trend for each condition. During IntCue, those who only had UP events adapted faster (*p-*value = 0.039). In contrast, during IntFB those who experienced both DOWN and UP events adapted faster than those who experienced only UP events (*p* < 0.000) (Table 5).

**TABLE 5:** Exploratory analysis on different responders. Index of adaptation *k* in response to UP events is reported as median (quartile 1– quartile 3) for IntCue and IntFB conditions among PDg.

***k* UP-events**	**only UP**	**UP+DOWN**	**Sign.**
IntCue	0.13 (0.10–0.18)	0.06 (0.02–0.17)	***p* = 0.039**
#deviators	#9	#5	
IntFB	0.10 (0.05–0.15)	0.28 (0.14–0.48)	***p* < 0.000**
#deviators	#8	#8	

*In the first column the group who received only UP events, while in the second column the group who received both UP and DOWN inputs during the 30 min of walking. Bold numbers indicate significant differences between groups.*

### Correlation Analysis

The participants’ SCOPA-Cog, MoCA, and MDS-UPDRS III scores did not correlate significantly with the median *k* rate during either condition or either task. However, as can be seen in Table 6, the median *k* rate correlated significantly with the number of deviations during the IntCue UP event (*r* = −0.57; *p-*value = 0.008), indicating that those with the slowest adaptations had the most deviations throughout the 30-minute walk. No significant correlations were observed for the IntCue DOWN events or any of the IntFB events.

**TABLE 6:** Correlation between the number of messages received (*#deviations*) and the median *k* rate of each subject during IntFB and IntCue conditions in response to UP-event and DOWN-event.

**Spearman’s *r***	**UP-event**	**DOWN-event**
IntFB	−0.15 (*p* = 0.525)	0.24 (*p* = 0.398)
#deviators	#21	#14
IntCue	**−0.57 (*p* = 0.008)**	0.13 (*p* = 0.731)
#deviators	#20	#10

*Spearman rank correlation coefficient *r* and relative *p*-value are shown in the table. Bold numbers indicate significant correlation.*

### Refractory Period During IntFB in PDg

Focusing on the PD group, the relative step constant τ = *1 / k*, with the *k* values reported in Table 3, indicates the refractory period of about seven steps for the UP event and three steps for the DOWN event during the IntFB condition. Higher values of τ are observed during the IntCue condition: about eight steps for the UP event and about five steps for the DOWN event. In Table 7 we report the values of τ.

**TABLE 7:** Refractory period. The relative step constant τ = *1 / k* [step] in the PD group.

***[step] (PD group)***	**UP event**	**DOWN event**
IntFB	6.7	3.0
IntCue	7.7	4.8

*τ represents the refractory period in response to UP and DOWN events and indicates the number of steps needed to adapt gait pattern and bring the cadence within the reference range following verbal/acoustic feedback.*

## Discussion

This study investigated the effects of intelligent auditory cueing (IntCue) and verbal feedback (IntFB) on motor adaptation in HE, PD, and PD subgroups (with and without FOG.) We introduced an innovative model to quantify motor adaptation speed following the two different acoustic messages. Thanks to our novel adaptation model, which applies a decay/growth exponential model to gait biofeedback for the first time, we can define the refractory period as the value of the relative step constant τ. This value indicates the steps needed to bring the cadence within the reference range following verbal/acoustic feedback.

Our results from the IntFB condition indicate a refractory period of about seven steps for the UP event and about three steps for the DOWN event for PD subjects. We found a similar (only slightly higher) refractory period for the IntCue condition. Clinicians and biofeedback developers designing novel therapeutic devices could apply our findings to determine the optimal timing for corrective feedback, optimizing gait rehabilitation while minimizing the risk of cue-dependency. In this way, the system could provide optimal corrective feedback in maintaining the proper gait pattern.

We hypothesized that the verbal and explicit nature of IntFB could speed up the motor response (Taylor et al., 2014), compared to IntCue which is more implicit and may thus requires some processing time to elaborate the direction of adaptation. In contrast to what assumed, our analysis could not detect different adaptations between IntFB and IntCue conditions. Nevertheless, in line with our expectations, the absolute median values of the decay/growth rate *k* for IntFB are larger than for IntCue in all groups and subgroups. This is consistent with the qualitative indications of the adaptation plots and is in line with the visual exploration performed in a previous study (Ginis et al., 2017a). Furthermore, when looking at the adaptation plots (Figure 3) it can be observed that there is an overshooting in IntFB only, which may be explained by the reference cadence indicated only during the IntCue condition.

We also expected that increasing the pace to the reference level would be a more difficult task than decreasing it. However, this trend is not confirmed within PDg by the statistical analysis. This lack of significance could be due to the small number of deviations recorded.

The group effect analyses yielded two results during IntCue: PDg had faster adaptation than HEg in response to UP events and FOG+g adapted faster than FOG-g in response to DOWN events. These results could be unexpected because HEg and FOG-g have better gait stability (fewer deviations) than PDg and FOG+g, respectively, as indicated in previous work (Ginis et al., 2017a, b). However, this is in line with previous work that showed a higher reliance on external input in PD compared to healthy subjects (Petzinger et al., 2013).

There were no differences in motor adaptation between the groups (HEg vs. PDg) or subgroups (FOG+g vs. FOG-g) during IntFB. In this regard, contradictory results can be found in literature. Roemmich et al. (2014) found that PD and HE adapted similarly, during the first strides after exposure to a split-belt gait pattern. On the other hand, Mohammadi et al. (2015) showed that FOG+ have more difficulties than FOG- and HE to adapt their gait to a split-belt treadmill over a short time period.

Our exploratory analysis revealed that during 30 min of walking, subjects who had only UP events adapted more slowly than those who had both UP and DOWN events during IntFB condition. This result is in agreement with our hypothesis: for those subjects who tend to slow down, it may be more difficult to increase their rhythm in response to corrective acoustic messages—compared to subjects who tend to both slow down and speed up. Instead, for IntCue we had the opposite trend, maybe because the metronome cues were more difficult to understand for those who received both up and down messages.

No significant correlations were found between our adaptation speed results and cognitive ability. In this protocol the subjects had to walk maintaining a determined cadence, without turning or avoiding obstacles. This steady state walking, with a reduced cognitive load, may be the reason for the lack of correlations found. In addition, the fact that all participants had an MMSE ≥ 24/30, conform the study’s inclusion criteria, suggests that further study is needed in a cohort with a wider cognitive spectrum. On the other hand, the original study found a correlation between gait stability (number of deviations) and the MoCA scale (with the same dataset) (Ginis et al., 2017a).

The relationship between our micro-analysis and the macro approach adopted in previous works (Ginis et al., 2017a, b) was evaluated by correlating the adaptation speed with the number of deviations. We found a negative trend only, during IntCue in response to UP events. During IntCue in response to DOWN events, as well as during IntFB, no significant correlations were observed. This finding seems to indicate that motor adaptation might be more effective during IntFB for all subjects, including those who require a lot of assistance through the intelligent messages. In fact, the previous work reported that subjects preferred verbal feedback (Ginis et al., 2017a). The negative trend found could also be explained by the slower adaptation leading to an increased number of deviations. In fact, participants may not yet be within the threshold values, triggering the feedback again. As expected, in response to DOWN events we did not find correlations in any conditions, suggesting that decreasing cadence is a relatively simple task which can be done fairly quickly.

A critical re-analysis of our results might suggest that an interesting solution could be the use of a combined-cue system, i.e., verbal feedback to increase or decrease cadence followed by the rhythmic cues to specify the target rate. This combined solution could trigger adaptation similar to the IntFB system, because of its explicit nature. On the other hand, due to the target rate indicated by the cueing, the combined-cue system may avoid the overshooting observed in part during the IntFB condition in the preliminary qualitative analysis. In any case, the joint use of IntFB and IntCue conditions could increase the overload of sensory and cognitive functions.

## Future Work

Effective tools for PD rehabilitation should allocate attention appropriately and lighten cognitive load (Stefan et al., 2005). The use of multisensory stimuli improves the learning process (Lehmann and Murray, 2005; Shams and Seitz, 2008), thanks to a reduced cognitive load and easier storage in short-term memory (von Kriegstein and Giraud, 2006; Schmitz et al., 2013). A multisensory approach also enhances perceptual processing (Shams and Seitz, 2008), known to be reduced in PD subjects with FOG (Davidsdottir et al., 2005). Mezzarobba et al. (2018) demonstrated the effectiveness of this approach in a study which used video and synthesized sounds to help PD subjects with FOG relearn gait movements and reduce freezing episodes.

Following this principle, it might be useful to add another sensory input to the IntFB condition. Proprioceptive feedback (such as vibrational stimuli) which require little or no cognitive processing or attention (Peterson and Smulders, 2015), might greatly improve motor adaptation in response to intelligent inputs. In fact, proprioceptive stimuli, in a closed loop system (Mancini et al., 2018), are already commonly used for gait rehabilitation in PD.

Future work also needs to address the long-term effect of gait rehabilitation on adaptation speed. It could be important to quantify the dynamics of adaptation during a trial and any possible motor-learning effect [i.e., the formation of a new motor pattern, in response to intelligent inputs, that occurs via long-term practice (Bastian, 2008)]. It would also be useful to evaluate motor adaptation through a prolonged, home-based training period, which would provide naturalistic data. Finally, it would be interesting to explore the use of different parameters (stride length, gait speed) instead of cadence to trigger the feedback.

It should be noted that further exploration of our model would benefit from ensuring that sufficient data are obtained to validate the qualitative and quantitative findings.

## Data Availability Statement

The datasets generated for this study are available on request to the corresponding author.

## Ethics Statement

The studies involving human participants were reviewed and approved by The local Ethics Committee of the KU Leuven and it was performed in accordance with the requirements of the International Council of Harmonization (Declaration of Helsinki, 1964). The patients/participants provided their written informed consent to participate in this study.

## Author Contributions

All authors listed have made a substantial, direct and intellectual contribution to the work, and approved it for publication.

## Conflict of Interest

AF (one of the founders) and LC (shareholder) have a significant financial interest in mHealth Technologies s.r.l., a company that may have a commercial interest in the results of this research. The remaining authors declare that the research was conducted in the absence of any commercial or financial relationships that could be construed as a potential conflict of interest.

## References

[B1] Ahn D. Chung H. Lee H. Kang K. Ko P. Kim N. S. (2017). Smart gait-aid glasses for parkinson’s disease patients. *IEEE Trans. Biomed. Eng.* 64 2394–2402. 10.1109/TBME.2017.2655344 28113199

[B2] Arnulfo G. Pozzi N. G. Palmisano C. Leporini A. Canessa A. Brumberg J. (2018). Phase matters: a role for the subthalamic network during gait. *PLoS One* 13:e0198691. 10.1371/journal.pone.0198691 29874298 PMC5991417

[B3] Bastian A. J. (2008). Understanding sensorimotor adaptation and learning for rehabilitation. *Curr. Opin. Neurol.* 21 628–633. 10.1097/WCO.0b013e328315a293 18989103 PMC2954436

[B4] Bohnen N. I. Jahn K. (2013). Imaging: what can it tell us about parkinsonian gait? *Mov. Disord.* 28 1492–1500. 10.1002/mds.25534 24132837 PMC3801220

[B5] Casamassima F. Ferrari A. Milosevic B. Ginis P. Farella E. Rocchi L. (2014). A wearable system for gait training in subjects with Parkinson’s disease. *Sensors* 14 6229–6246. 10.3390/s140406229 24686731 PMC4029669

[B6] Chong R. Hyun Lee K. Morgan J. Mehta S. (2011). Closed-loop vr-based interaction to improve walking in parkinson’s disease. *J. Nov. Physiother.* 1 1–7. 10.4172/2165-7025.1000101

[B7] Davidsdottir S. Cronin-Golomb A. Lee A. (2005). Visual and spatial symptoms in Parkinson’s disease. *Vis. Res.* 45 1285–1296. 10.1016/j.visres.2004.11.006 15733961

[B8] Espay A. J. Baram Y. Dwivedi A. K. Shukla R. M. Gartner M. Gaines L. (2010). At-home training with closed-loop augmented-reality cueing device for improving gait in patients with Parkinson disease. *J. Rehabil. Res. Dev.* 47 573–581. 20848370 10.1682/jrrd.2009.10.0165

[B9] Ferrari A. Ginis P. Nieuwboer A. Greenlaw R. Muddiman A. Chiari L. (2016). “Handling gait impairments of persons with parkinson’s disease by means of real-time biofeedback in a daily life environment,” in *Inclusive Smart Cities and Digital Health*, eds Chang C. K. Chiari L. Cao Y. Jin H. Mokhtari M. Aloulou H. (Cham: Springer International Publishing), 250–261. 10.1007/978-3-319-39601-9_22

[B10] Ferrazzoli D. Ortelli P. Madeo G. Giladi N. Petzinger G. M. Frazzitta G. (2018). Basal ganglia and beyond: the interplay between motor and cognitive aspects in parkinson’s disease rehabilitation. *Neurosci. Biobehav. Rev.* 90 294–308. 10.1016/j.neubiorev.2018.05.007 29733882

[B11] Gill D. J. Freshman A. Blender J. A. Ravina B. (2008). The Montreal cognitive assessment as a screening tool for cognitive impairment in Parkinson’s disease. *Mov. Disord.* 23 1043–1046. 10.1002/mds.22017 18381646

[B12] Ginis P. Heremans E. Ferrari A. Bekkers E. M. J. Canning C. G. Nieuwboer A. (2017a). External input for gait in people with Parkinson’s disease with and without freezing of gait: one size does not fit all. *J. Neurol.* 264 1488–1496. 10.1007/s00415-017-8552-6 28653213

[B13] Ginis P. Heremans E. Ferrari A. Dockx K. Canning C. G. Nieuwboer A. (2017b). Prolonged walking with a wearable system providing intelligent auditory input in people with parkinson’s disease. *Front. Neurol.* 8:128 10.3389/fneur.2017.00128 PMC5382170 28428770

[B14] Ginis P. Nieuwboer A. Dorfman M. Ferrari A. Gazit E. Canning C. G. (2016). Feasibility and effects of home-based smartphone-delivered automated feedback training for gait in people with Parkinson’s disease: a pilot randomized controlled trial. *Parkinsonism Relat. Disord.* 22 28–34. 10.1016/j.parkreldis.2015.11.004 26777408

[B15] Goetz C. G. Tilley B. C. Shaftman S. R. Stebbins G. T. Fahn S. Martinez-Martin P. (2008). movement disorder society-sponsored revision of the unified parkinson’s disease rating scale (mds-updrs): scale presentation and clinimetric testing results. *Mov. Disord.* 23 2129–2170. 10.1002/mds.22340 19025984

[B16] Hausdorff J. M. (2009). Gait dynamics in Parkinson’s disease: common and distinct behavior among stride length, gait variability, and fractal-like scaling. *Chaos* 19:026113. 10.1063/1.3147408 19566273 PMC2719464

[B17] Lee S. J. Yoo J. Y. Ryu J. S. Park H. K. Park H. K. Chung S. J. (2012). The effects of visual and auditory cues on freezing of gait in patients with Parkinson disease. *Am. J. Phys. Med. Rehabil.* 91 2–11. 10.1097/PHM.0b013e31823c7507 22157432

[B18] Lehmann S. Murray M. M. (2005). The role of multisensory memories in unisensory object discrimination. *Brain Res. Cogn. Brain Res.* 24 326–334. 10.1016/j.cogbrainres.2005.02.005 15993770

[B19] Mancini M. Smulders K. Harker G. Stuart S. Nutt J. G. (2018). Assessment of the ability of open- and closed-loop cueing to improve turning and freezing in people with Parkinson’s disease. *Sci. Rep.* 8:12773. 10.1038/s41598-018-31156-4 30143726 PMC6109152

[B20] Marinus J. Visser M. Verwey N. A. Verhey F. R. J. Middelkoop H. A. M. Stiggelbout A. M. (2003). Assessment of cognition in Parkinson’s disease. *Neurology* 61 1222–1228. 14610124 10.1212/01.wnl.0000091864.39702.1c

[B21] Mazzoni P. Shabbott B. Cortés J. C. (2012). Motor control abnormalities in Parkinson’s disease. *Cold Spring Harb. Perspect. Med.* 2:a009282. 10.1101/cshperspect.a009282 22675667 PMC3367543

[B22] Mezzarobba S. Grassi M. Pellegrini L. Catalan M. Kruger B. Furlanis G. (2018). A novel therapeutic protocol for parkinson’s patient with freezing of gait. *Front. Neurol.* 8:723. 10.3389/fneur.2017.00723 29354092 PMC5758544

[B23] Mohammadi F. Bruijn S. M. Vervoort G. van Wegen E. E. Kwakkel G. Verschueren S. (2015). Motor switching and motor adaptation deficits contribute to freezing of gait in Parkinson’s disease. *Neurorehabil. Neural Repair* 29 132–142. 10.1177/1545968314545175 25416741

[B24] Morris M. E. (2006). Locomotor training in people with Parkinson disease. *Phys. Ther.* 86 1426–1435. 10.2522/ptj.20050277 17012646

[B25] Morris M. E. Iansek R. Galna B. (2008). Gait festination and freezing in Parkinson’s disease: pathogenesis and rehabilitation. *Mov. Disord.* 23 (Suppl. 2), S451–S460. 10.1002/mds.21974 18668618

[B26] Morris M. E. Martin C. L. Schenkman M. L. (2010). Striding out with Parkinson disease: evidence-based physical therapy for gait disorders. *Phys. Ther.* 90 280–288. 10.2522/ptj.20090091 20022998 PMC2816030

[B27] Nieuwboer A. Rochester L. Müncks L. Swinnen S. P. (2009). Motor learning in parkinson’s disease: limitations and potential for rehabilitation. *Parkinsonism Relat. Disord.* 15 S53–S58. 10.1016/S1353-8020(09)70781-3 20083008

[B28] Peterson D. S. Smulders K. (2015). Cues and attention in parkinsonian gait: potential mechanisms and future directions. *Front. Neurol.* 6:255 10.3389/fneur.2015.00255 PMC4672041 26696955

[B29] Petzinger G. M. Fisher B. E. McEwen S. Beeler J. A. Walsh J. P. Jakowec M. W. (2013). Exercise-enhanced neuroplasticity targeting motor and cognitive circuitry in parkinson’s disease. *Lancet Neurol.* 12 716–726. 10.1016/S1474-4422(13)70123-6 23769598 PMC3690528

[B30] Pozzi N. G. Canessa A. Palmisano C. Brumberg J. Steigerwald F. Reich M. M. (2019). Freezing of gait in Parkinson’s disease reflects a sudden derangement of locomotor network dynamics. *Brain* 142 2037–2050. 10.1093/brain/awz141 31505548 PMC6598629

[B31] Redgrave P. Rodriguez M. Smith Y. Rodriguez-Oroz M. C. Lehericy S. Bergman H. (2010). Goal-directed and habitual control in the basal ganglia: implications for Parkinson’s disease. *Nat. Rev. Neurosci.* 11 760–772. 10.1038/nrn2915 20944662 PMC3124757

[B32] Rispens S. M. van Schooten K. S. Pijnappels M. Daffertshofer A. Beek P. J. van Dieën J. H. (2015). Do extreme values of daily-life gait characteristics provide more information about fall risk than median values? *JMIR Res. Protoc.* 4:e4. 10.2196/resprot.3931 25560937 PMC4296095

[B33] Roemmich R. T. Nocera J. R. Stegemöller E. L. Hassan A. Okun M. S. Hass C. J. (2014). Locomotor adaptation and locomotor adaptive learning in Parkinson’s disease and normal aging. *Clin. Neurophysiol.* 125 313–319. 10.1016/j.clinph.2013.07.003 23916406 PMC3844121

[B34] Sánchez-Ferro Á Maetzler W. (2016). Advances in sensor and wearable technologies for Parkinson’s disease. *Mov. Disord.* 31:1257. 10.1002/mds.26746 27477675

[B35] Schmitz G. Mohammadi B. Hammer A. Heldmann M. Samii A. Münte T. F. (2013). Observation of sonified movements engages a basal ganglia frontocortical network. *BMC Neurosci.* 14:32. 10.1186/1471-2202-14-32 23496827 PMC3602090

[B36] Shams L. Seitz A. R. (2008). Benefits of multisensory learning. *Trends Cogn. Sci.* 12 411–417. 10.1016/j.tics.2008.07.006 18805039

[B37] Shine J. M. Handojoseno A. M. A. Nguyen T. N. Tran Y. Naismith S. L. Nguyen H. (2014). Abnormal patterns of theta frequency oscillations during the temporal evolution of freezing of gait in Parkinson’s disease. *Clin. Neurophysiol.* 125 569–576. 10.1016/j.clinph.2013.09.006 24099920

[B38] Spildooren J. Vercruysse S. Meyns P. Vandenbossche J. Heremans E. Desloovere K. (2012). Turning and unilateral cueing in Parkinson’s disease patients with and without freezing of gait. *Neuroscience* 207 298–306. 10.1016/j.neuroscience.2012.01.024 22285883

[B39] Stefan K. Cohen L. G. Duque J. Mazzocchio R. Celnik P. Sawaki L. (2005). Formation of a motor memory by action observation. *J. Neurosci.* 25 9339–9346. 10.1523/JNEUROSCI.2282-05.2005 16221842 PMC6725701

[B40] Tard C. Delval A. Devos D. Lopes R. Lenfant P. Dujardin K. (2015). Brain metabolic abnormalities during gait with freezing in Parkinson’s disease. *Neuroscience* 307 281–301. 10.1016/j.neuroscience.2015.08.063 26341909

[B41] Taylor J. A. Krakauer J. W. Ivry R. B. (2014). Explicit and implicit contributions to learning in a sensorimotor adaptation task. *J. Neurosci.* 34 3023–3032. 10.1523/JNEUROSCI.3619-13.2014 24553942 PMC3931506

[B42] Vercruysse S. Spildooren J. Heremans E. Vandenbossche J. Wenderoth N. Swinnen S. P. (2012). Abnormalities and cue dependence of rhythmical upper-limb movements in Parkinson patients with freezing of gait. *Neurorehabil. Neural Repair* 26 636–645. 10.1177/1545968311431964 22291041

[B43] von Kriegstein K. Giraud A.-L. (2006). Implicit multisensory associations influence voice recognition. *PLoS Biol.* 4:e326. 10.1371/journal.pbio.0040326 17002519 PMC1570760

